# Field efficacy of Febantel, Pyrantel embonate and Praziquantel (Drontal® Tasty) against naturally acquired intestinal helminths of hunting dogs in southern Italy

**DOI:** 10.1186/s13071-025-07027-z

**Published:** 2025-09-24

**Authors:** Farwa Humak, Francesco Buono, Vincenzo Veneziano, Diego Piantedosi, Elisa Castaldo, Stefano Scarcelli, Francesco Locantore, Anna Paola Rivolta, Stefania Rotondi, Norbert Mencke, Katrin Blazejak

**Affiliations:** 1 Department of Veterinary Medicine and Animal Productions, University of Naples Federico II, Naples, Italy; 2 Department of Preventive Medicine, ASL Foggia SIAV A Nord, Foggia, Italy; 3 Vetoquinol Italia Srl, Forlì, Italy; 4 Vetoquinol S.A, Paris, France

**Keywords:** Anthelmintic treatment, Drontal^®^, Helminth infection, Hunting dogs, Italy

## Abstract

**Background:**

Dogs in rural areas and hunting dogs in particular, are at higher risk of intestinal helminth infections compared with family dogs. Thus, certain management practices including faecal/coprological analysis, implementing regular deworming protocols, post-treatment evaluations and high-quality hygiene are required in hunting dogs to maintain their health and activity. The aim of this study was to evaluate the efficacy of Drontal^®^ Tasty (Vetoquinol S.A.) for the treatment of gastrointestinal helminths in naturally infected hunting dogs in Italy during the hunting and non-hunting season.

**Methods:**

Hunting dogs (*n* = 387) faecal samples were collected from Campania and Basilicata regions, and study was divided into two phases: hunting (P1) and non-hunting (P2) periods. Each sample was screened using Mini-FLOTAC technique with ZnSO_4_ as flotation medium (SG: 1.350). The dogs (*n* = 142) were enrolled on the basis of pre-screening faecal egg counts at day 0 of P1. Dogs exhibiting ≥ 50 EPG species were treated on day 0 of P1 and sampled on day 0, 7 and 14 for faecal analysis. Dogs enrolled in P1 were again controlled in P2 (*n* = 128) regardless of EPG following the same scheme of P1. The primary criterion for treatment efficacy was the reduction of faecal egg count after D7 and D14 in both P1 and P2.

**Results:**

The anthelmintic efficacy of Drontal^®^ Tasty against *Toxocara canis* was 99.7% and 100% at day 14 in P1 and P2, respectively; for *Toxascaris leonina* 100% at day 14 in P1, for Ancylostomatidae 100% at day 14 in both P1 and P2; for *Trichuris vulpis* 88.6% and 99.8% at day 14 in P1 and P2, respectively. For *Dipylidium caninum* and Taeniidae no positive dogs were detected at day 14 in P1. Although drug is off label for the control of *Capillaria* spp. the Faecal Egg Count Reduction ranging from 42.1% to 84.5% in P1 and P2, respectively.

**Conclusions:**

Drontal^®^ Tasty was well-tolerated and safe against major nematodes and cestodes in hunting dogs. It is the optimum choice to treat helminth infection in hunting dogs under field condition.

**Graphical Abstract:**

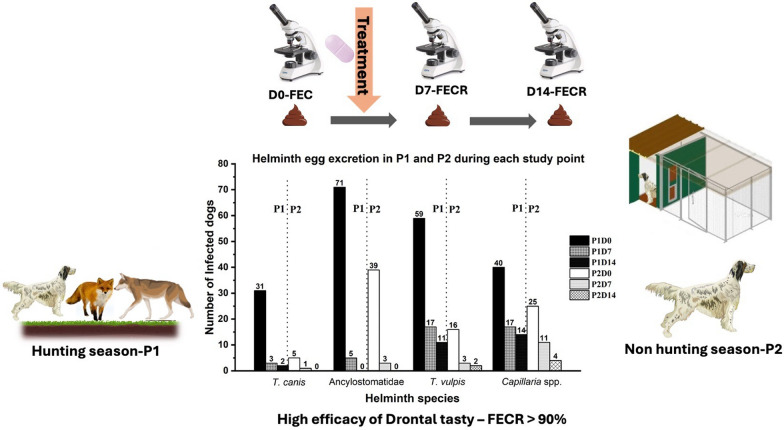

**Supplementary Information:**

The online version contains supplementary material available at 10.1186/s13071-025-07027-z.

## Background

Helminthic infections, particularly gastrointestinal helminths, represent a significant and prevalent group of diseases encountered in companion animal clinical practice worldwide [1]. Besides clinical relevant infections, especially in puppies and young dogs, the major helminths of dogs are of serious concerns due to their zoonotic potential [2].

Dogs (*Canis lupus familiaris*) represent the most versatile domesticated pets worldwide over 15,000 years [3]. Canids can act as reservoirs and carriers for several intestinal helminths some of that with zoonotic concern [4, 5] and dogs involved in hunting practices in rural and sylvatic environments [6], in close contact with wild canids (i.e. red fox – *Vulpes vulpes*, golden jackal – *Canis aureus* and wolf – *Canis lupus*), could facilitate the transmission and diffusion of several zoonotic parasites.

These infections not only pose health risks to the dogs themselves but also have broader ecological and sanitary implications [7–9]. For instance, nematodes such as the hookworms *Ancylostoma caninum*, *Uncinaria stenocephala* and the roundworm *Toxocara canis*, are commonly found in dogs and have zoonotic potential, causing diseases in humans such as cutaneous and visceral larva migrans syndromes. Their impact extends to other clinical presentations such as eosinophilic enteritis (especially with *A. caninum*), ocular larva migrans which can lead to vision impairment or blindness, and in some cases, neurological signs including eosinophilic meningoencephalitis due to larval migration to the central nervous system (particularly with *Toxocara* spp*.*). These infections also contribute to conditions like chronic abdominal pain and allergic manifestations, highlighting their broader public health significance [10].

Even though several studies indicated a decline in helminth infections in dog population across Europe [11], attributed to several factors, such as enhanced hygiene practices, increased awareness on parasitic diseases among pet owners, improved socioeconomic status and the use of broad-spectrum anthelmintics, helminths continue to represent a significant global health concern in Europe due to their occurrence in endemic areas [12]. These helminth infections are evident in high-risk exposed dogs (e.g. stray and hunting dogs) correlated to a poor management practice and lower frequency of veterinary consultation [8]. As an example, the findings of a survey in hunting dogs in southern Italy, showed high prevalence (56.6%) of overall intestinal helminths, commonest were hookworms (39.3%), followed by *Capillaria aerophila* (20.5%), *Trichuris vulpis* (12.6%) and *T. canis* (11.3%) [13].

Hunting dogs are at an increased risk of helminth infections during the hunting season due to exposure to parasitic stages in contaminated environments shared with wildlife [13]. In contrast, during the non-hunting season, the risk of parasitic infection is lower, as dogs are trained in areas where the contact with wild animals is limited.

Consequently, certain management practices are now considered as the way forward to control of main canine helminth infections to protect both animal and human health, including the treatment with licensed anthelmintics based on faecal examination, implementing regular deworming protocols and monitoring programs [14]. The frequency of deworming mostly depends on the legislation in the respective country, the prescription of veterinary professionals, perception of owners and most importantly individual risk assessments for dogs (www.esccap.org). For example, hunting dogs could be at major risk of infection by zoonotic pathogens including parasites as well as reported in companion animals fed raw meat (e.g. Bones and Raw Food—BARF) [15, 16].

Several active anthelmintic compounds, such as febantel, pyrantel embonate and praziquantel, are administered in dogs for controlling intestinal helminths infection [17]. Febantel, a prodrug, is metabolised to fenbendazole and oxfendazole, thus exhibiting similar efficacy and a comparable mode of action to other benzimidazoles. Febantel demonstrates higher efficacy against hook- and roundworms; it is effective also against the whipworm *T. vulpis*, though its efficacy against immature stages is poor [18]. Pyrantel, a tetrahydropyrimidine, has a broad-spectrum nematocide activity, exhibiting an efficacy rate of 90–100% against hook- and roundworms but no activity against *Trichuris* spp. [19]. Moreover, febantel and pyrantel have moderate activity against the developing larval stages of hookworms [20]. Praziquantel, an isoquinolone, is the drug of choice against a wide range of adult and larval stages of cestodes and trematodes in both humans as well as animals [21].

The efficacy of Drontal^®^ Plus (febantel, pyrantel embonate and praziquantel) against intestinal parasites and its safe use in dogs have been documented in previous studies [22, 23]. In companion animals, unlike ruminants and horses, anthelmintic resistance (AHR) is uncommon; however, the first report of AHR in canine hookworms to the pyrimidine was reported 20 years ago in Australia [24] whereas resistance to fenbendazole was firstly reported in USA in 2019 [25]. Moreover, some recent reports highlight the spread of resistance in canine hookworms both in Australia [26] and in North America [27, 28]. Concerning *T. canis,* to the best of our knowledge, to date, only one confirmed case of AHR was reported in a greyhound breeding farm in the USA [29]. With regards to cestodes, AHR in *Dipylidium caninum* to praziquantel has been reported in USA [30].

Considering the recent reports of AHR in dog population, the aims of this field study were to re-evaluate the efficacy and safety of the combination of febantel, pyrantel embonate and praziquantel (Drontal^®^ Tasty) after 10 years from its introduction in the market for the treatment of gastrointestinal helminths in naturally infected hunting dogs in Italy and to evaluate the treatment scheme during the hunting and non-hunting seasons.

## Methods

### Study setting

The study was conducted from October 2023 to September 2024 in two southern Italy regions (Campania and Basilicata).

The study was structured into two phases: the first part of the study, Phase 1 (P1) was scheduled during the hunting season from October 2023 to January 2024. The second part, Phase 2 (P2) covered the non-hunting season from April 2024 to September 2024.

### Experimental design

The research was designed in compliance with the following internationally accepted guidelines: International Cooperation on Harmonisation of Technical Requirements for Registration of Veterinary Medicinal Products-VICH GL7, ‘Efficacy of Anthelmintics: General Requirements’ [31]; ‘Efficacy of Anthelmintics: Specific Recommendations for Canines’-VICH GL19 [32]; ‘World Association for the Advancement of Veterinary Parasitology (WAAVP): Second edition of guidelines for evaluating the efficacy of anthelmintics for dogs and cats’ [33].

All procedures on the studied dogs were performed with the owner’s consent and the investigation was approved by the Ethical Animal Care and Use Committee of the University of Naples ‘Federico II’ (protocol number PG/2022/0146451). The study was a non-blinded, non-controlled and non-randomised clinical field trial including privately owned dogs. All owners were informed of the treatment assignment of this trial and signed an informed consent sheet.

### Parasitological screening and clinical examination of dogs

The dogs were examined at private veterinary clinics located in the territory of the study area. Dogs having good health status, irrespective of breed and sex, with a minimum age of 6 months, a minimum body weight > 2.0 kg, that hadn’t received anthelminthic treatment in the previous 12 weeks and with evidence of intestinal helminth infections (Faecal Egg Count—FEC ≥ 50 Eggs per Gram—EPG) as *T. canis*, *Toxascaris leonina*, Ancylostomatidae, *T. vulpis,* or positive for *D. caninum* and Taeniidae were eligible for inclusion in Phase 1 of the study. Each dog enrolled and treated in P1 was retested in P2 and enrolled in case of a positive FEC irrespective of EPG count.

Exclusion criteria were any dogs suffering of other disease or injuries that could interfere with the study. Likewise, pregnant or lactating females during the study period were not enrolled.

For each included dog, data such as sex, age and living area (urban and rural) were recorded.

### Faecal examinations

Individual faecal samples were collected and placed in plastic bags, which were labelled with the dogs ID, owner name, date and location of sampling. The faecal samples were examined first macroscopically for the presence of parasitic elements (e.g. proglottids). Coprological examinations were performed by investigators directly at the private veterinary clinics or at the laboratory of Parasitology and Parasitic Diseases at the University of Naples ‘Federico II’. The faecal samples were refrigerated during transport and examined within 48 h. FEC was performed by using Mini-FLOTAC technique, with a detection limit of 5 EPG of faeces with a zinc sulphate (ZnSO_4_) as floatation solution (specific gravity 1.350) [34]. For cestodes, only qualitative data (positive/negative) were reported [35]. In cases that FEC was performed directly in private veterinary clinics and dogs tested positive, the anthelmintic treatment was administered immediately. Conversely, when the FEC was performed in the laboratory of University of Naples ‘Federico II’, the anthelmintic treatment was administered within 24–48 h. In both cases, anthelmintic treatment was carried out by investigators.

An individual FEC was performed for each dog on day 7 (P1D7) post treatment; if the FEC was negative, dogs ended the P1, if the FEC remained positive, a second coprological examination was conducted at 14 days post-treatment (P1D14) with the remaining dogs terminating P1. Dogs enrolled and treated during P1 were suitable for inclusion in P2. In case of a positive FEC in P2, irrespective of the EPG, the dogs received a second treatment with Drontal^®^ Tasty according to the same scheme as in P1. Anthelmintic efficacy was consecutively evaluated on day 7 (P2D7) and, where applicable, on day 14 (P2D14).

### Clinical examination

The dogs were clinically examined at each sampling time by the same veterinarian investigators (VV, DP, FB) upon enrolment (before the treatment) and at the end of the individual study period (D7 and/or D14) when the dogs were presented for the post-treatment clinical and coprological examinations.

Following treatment, each dog was observed for a duration of 30 min for vomiting or any other adverse events. Furthermore, the dogs were monitored until the termination of each respective study phase (P1 and P2) for a maximum of 14 days post-treatment. The owners were informed by investigators about adverse events monitoring and invited to signal them promptly in case of any adverse reaction.

### Anthelmintic treatment

Prior to the anthelmintic administration (day 0; P1 and P2), each dog was weighed using a scale. The treatment was performed once orally, with a minimum dose of 15 mg febantel/kg body weight (BW), 14.4 mg pyrantel embonate/kg BW and 5 mg praziquantel/kg BW (equivalent to one tablet Drontal^®^ Tasty per 10 kg/BW, Vetoquinol S.A., Lure, France) according to their body weight and the label instructions. Dogs were kept with their owners and receive the same housing, diet and ration food before, during and after the study. Water was administered ad libitum.

The study dogs did not receive any additional medication during the entire study period.

### Efficacy calculation

The efficacy against *T. canis*, *T. leonina*, Ancylostomatidae and *T. vulpis*, was evaluated in terms of faecal egg count reduction (FECR) between the first faecal sample (day 0) and the two faecal samples obtained post treatment (days 7 and 14), according to the World Association for Advancement of Veterinary Parasitology Guidelines [33].

The threshold efficacy was set at ≥ 90%, as determined by the following formula:

$$FECR= \frac{{EPG}_{pre-treatment}-{EPG}_{post-treatment}}{{EPG}_{pre-treatment}}x 100$$

The efficacy of treatment against cestodes (*D. caninum* and Taeniidae) was assessed descriptively by comparing the number of positive dogs (i.e. those with the presence of ovigerous capsules/eggs at copromicroscopic examination and/or visual presence of proglottids in faeces) before and after treatment [25].

Dogs positive to *Capillaria* spp. were not included in the evaluation of anthelmintic efficacy, but the FEC were recorded.

### Statistical analysis

The data were supplied in electronic form (MS Excel) for the statistical analysis. The data for analysis were transferred to the analysis programs according to idv-SOPs by means of the validated idv Excel-transfer. The analysis was performed with the validated program Testimate Version 6.5 from IDVD, Gauting (validation of software, hardware and user according to FDA 21 CFR Part 11). All criteria were analysed descriptively. The egg counts were described by number of valid cases, mean, median, standard deviation, quartiles, minimum and maximum. A Student's *t* test was carried out to compare the intensity of infection (mean EPG count) during the two study phases (P1 and P2). Values of *p* < 0.05 were considered significant.

## Results

### Study dogs

A total of 387 hunting dogs (327; 84.5% from Campania and 60; 15.5% from Basilicata) were screened for the presence of intestinal helminths throughout coprological examinations.

Out of 387 dogs screened for inclusion in the study, 142 were enrolled in P1, showing a FECs ≥ 50 EPG for at least one of the targeted parasites. The total infection rate and mean EPG are outlined in Table 1.

**Table 1:** Overall infection rate and range of faecal egg counts of 142 hunting dogs included in the study during the hunting season (Phase 1)

Helminths	Infection rate		Mean EPG ± SD	EPG–Range
	Number of positive dogs (N)	Prevalence (%) (95% CI)		
*Toxocara canis*	33	23.2 (16.3–30.2)	172.12 ± 208.14	5–895
*Toxoascaris leonina*	1	0.7 (0–2.1)	n.a	n.a.*
Ancylostomatidae	79	55.6 (47.5–63.8)	193.23 ± 226.80	5–980
*Trichuris vulpis*	67	47.2 (39.0–55.4)	95.30 ± 169.39	5–1200
*Capillaria* spp.	43	30.3 (22.7–37.8)	95.00 ± 175.69	5–920
*Dipylidium caninum*	23	16.2 (10.1–22.3)	n.a	n.a
Taeniidae	5	3.5 (0.5–6.6)	n.a	n.a

*EPG* egg per gram of faeces, *n.a*. not applicable; n.a.*: only one dog was positive for *T. leonina* eggs showing an EPG count of 2,110

The demographic of included dogs is outlined below: 48/142 (33.8%) were juveniles (< 2 years), 83/142 (58.5%) adults (2–7 years) and 11/142 (7.7%) mature adults (> 7 years); 81/142 (57.1%) were male while 61/142 (42.9%) female. The majority of the dogs, 120/142 (84.5%), resided in rural areas while 22/142 (15.5%) in urban areas.

Out of the 142 dogs included in P1, 128/142 (90.1%) were screened for the second time in P2, whereas 14/142 (9.9%) were not available for following reasons: 5/14 (35.7%) for owner’s unavailability, 2/14 (14.3%) dogs were pregnant, 3/14 (21.4%) dogs were lactating, 2/14 (14.3%) dogs were sold and 2/14 (14.3%) dogs died.

### Faecal egg count and anthelmintic efficacy—Phase 1 (Hunting season)

In the hunting season (P1) at study day 7 (D7), out of 142 treated dogs, 22 (15.5%) remained positive for at least one helminth species after the treatment. Single infection was reported in 14/22 dogs (63.6%), while mixed infections were found in 8/22 dogs (36.4%).

The anthelmintic efficacy on D7 was 99.2% for *T. canis*, 100% for *T. leonina*, 99.6% for Ancylostomatidae and 81.7% for *T. vulpis*, respectively. Out of the 23/142 (16.2%) dogs tested positive to *D. caninum* on D0 none was tested positive after treatment on D7. A total of 5 (3.5%) dogs were tested positive for Taeniidae eggs on D0 and only 1 dog (20.0%) remained positive on D7 after treatment.

At study day 14 (D14), 14/22 (63.6%) dogs were still positive whereas 8/22 (24.4%) were negative. Out of these, 3/14 (21.4%) were positive only to *Capillaria* spp. Of the remaining 11 positive dogs, single infection was reported in 8/11 dogs (72.7%) while mixed infection in 3/11 dogs (27.3%).

The anthelmintic efficacy at D14 was 99.7% for *T. canis*, 100% for *T. leonina,* 100% for Ancylostomatidae and 88.6% for *T. vulpis.* The dogs which was still positive for Taeniidae on D7 was tested negative on D14 as well.

Regarding *Capillaria* spp. on D7 and D14, FECR was 68.7% and 42.1% respectively.

### Faecal egg count and anthelmintic efficacy—Phase 2 (Non-Hunting season)

A total of 128 dogs of study group P2 were again screened on D0. Out of this, 69/128 (53.9%) showed a negative FEC and thus were not included in P2, whereas 59/128 (46.1%) tested positive for at least one of the targeted parasites were included. Single infection was reported in 37/59 dogs (62.7%) while mixed infection in 22/59 dogs (37.3%). Out of 59 positive dogs, 8 (13.5%) were found to be positive only for *Capillaria* spp. and were therefore not included in P2. Overall, 51/128 (39.8%) were re-treated once with Drontal^®^ Tasty.

On D7, out of 51 dogs treated, 39 (76.5%) had a negative FEC (FEC = 0), 6 dogs (11.8%) were positive only to *Capillaria* spp. thus they were not screened on day 14 and completed P2; 6 dogs (11.8%) were again tested on D14.

On D7 FECR was 86.7% for *T. canis*, and 94.3% for Ancylostomatidae; whereas, for *T. vulpis* it was 0%.

On D14, 2/6 (33.3%) dogs had a negative FEC (FEC = 0), while 4/6 (66.7%) dogs were positive with a single infection in 2/4 (50.0%) dogs and mixed infection in other 2/4 (50.0%) dogs. The anthelmintic efficacy (D14) in terms of reduction of faecal egg counts was 100% for *T. canis*, 100% for Ancylostomatidae and 99.8% for *T. vulpis*.

In Table 2, the number of positive dogs and the anthelmintic efficacy are summarized for both P1 and P2 at each study point.

**Table 2:** Number of positive dogs excreted helminth parasite stage on each study point and anthelmintic efficacy (% FECR)

Helminth parasite stage	Study phase*	D0 (*n* positive dogs)	D7 (*n* positive dogs) (FECRT %)	D14 (*n* positive of dogs) (FECRT %)
*Toxocara canis* eggs	1	33	3 (99.2%)	2 (99.7%)
	2	5	1 (86.7%)	0 (100%)
*Toxascaris leonina* eggs	1	1	0 (100%)	0 (100%)
	2	0	–	–
Ancylostomatidae eggs	1	79	5 (99.6%)	0 (100%)
	2	38	3 (94.3%)	0 (100%)
*Trichuris vulpis* eggs	1	67	18 (81.7%)	11 (88.6%)
	2	16	3 (0%)	2 (99.8%)
*Capillaria* spp. eggs	1	43	21 (68.7%)	17 (42.1%)
	2	25	11 (42.3%)	4 (84.2%)
*Dipylidium caninum* eggs/proglottids	1	23	0	0
	2	0	0	0
Taeniidae eggs/proglottids	1	5	1	0
	2	0	0	0

^*^Study phase P1 = hunting season; study phase P2 = non-hunting-season

The FECR for *Capillaria* spp. was 42.3% and 84.2% at D7 and D14, respectively.

### Comparison between P1 (Hunting) and P2 (Non-Hunting Season)

To compare the prevalence of helminth infections during the hunting (P1) and non-hunting (P2) season, only the same dogs (n. 128) that had been included in both study phases were compared. Statistical difference (*p* < 0.001) between P1 and P2 was detected only for Ancylostomatidae and *T. vulpis.*

At D0, Ancylostomatidae eggs were reported in 71/128 (P1) (mean ± standard deviation, 205.3 ± 236.1) and 39/128 dogs (P2) (mean ± standard deviation, 115.5 ± 170.4), respectively; reporting a higher frequency and intensity of infection in P1.

**Fig. 1:**
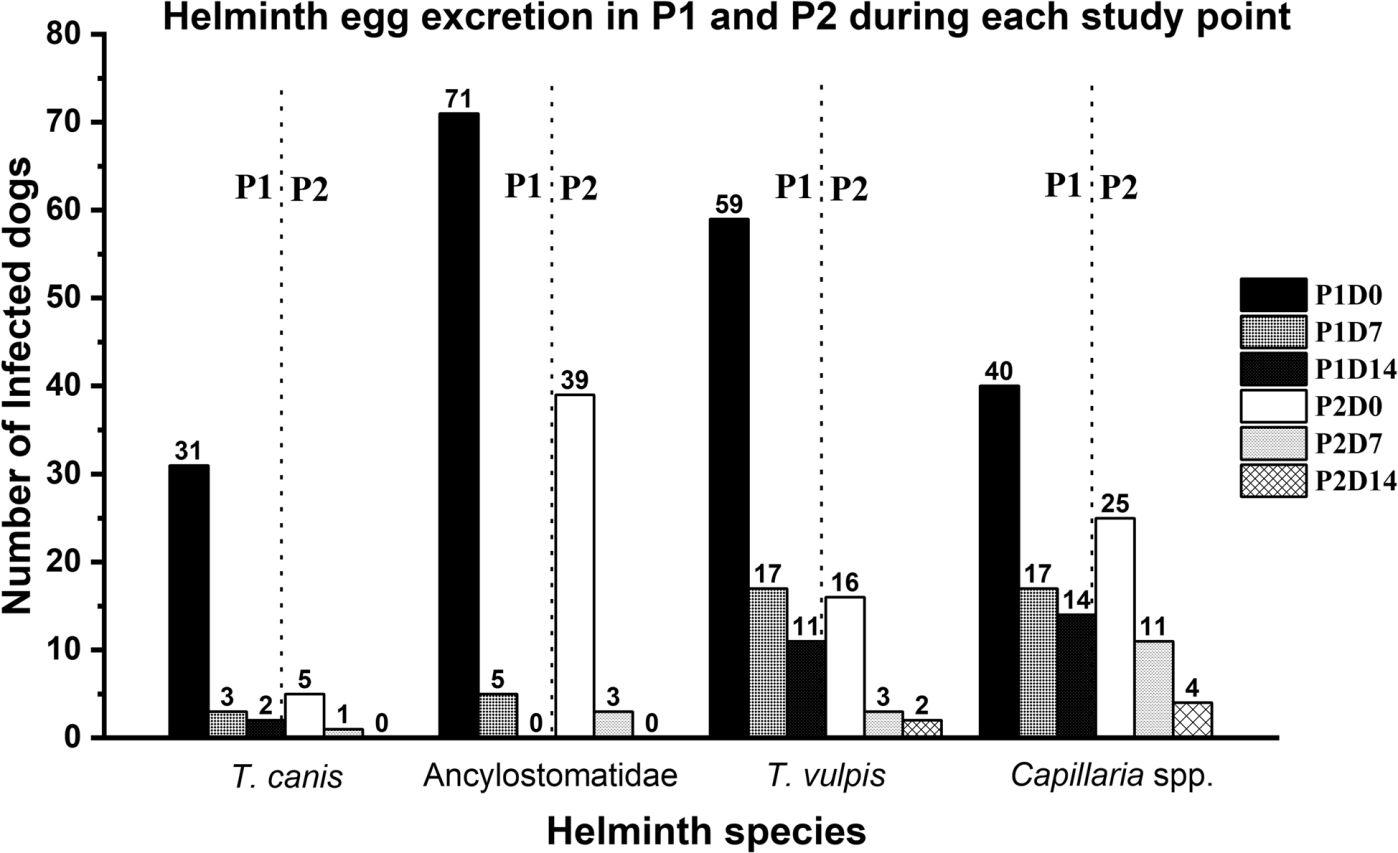
Comparison between hunting- (P1) and non-hunting-season (P2) on 128 enrolled dogs

Concerning *T. vulpis* at D0, eggs were observed in 59/128 dogs (P1) (mean ± standard deviation, 99.8 ± 178.7) and 16/128 dogs (P2) (mean ± standard deviation, 255.0 ± 463.1), respectively. The frequency of infection was lower in P2 than P1, however the intensity was higher in P2 (Table 3; Fig. 1).

**Table 3:** Comparison of helminth infection in hunting (P1) and non-hunting (P2) seasons in 128 enrolled dogs: number of positive dogs and mean EPG count ± standard deviation (SD)

Helminth species	Study phase	D0		D7		D14	
		*N* (Mean ± Std. Dev.)	*p* Value	*N* (Mean ± Std. Dev.)	*p* Value	*N* (Mean ± Std. Dev.)	*p* Value
*T. canis*	1	31 (177.58 ± 213.56)	0.2	3 (55.00 ± 34.64)	–	2 (45 ± 49.5)	–
	2	5 (55.00 ± 59.9)		1 (30)		0	
*T. leonina*	1	1 (2,110)	–	0	–	0	–
	2	0		0		0	
Ancylostomatidae	1	71 (205.28 ± 236.07)	0.03*	5 (9.00 ± 5.48)	0.1	0	–
	2	39 (115.51 ± 170.43)	–	3 (28.33 ± 23.09)	–	0	–
*T. vulpis*	1	59 (99.83 ± 178.72)	0.04*	17 (15.59 ± 19.83)	0.01*	11 (14.55 ± 8.2)	0.9
	2	16 (255 ± 463.13)		3 (141.67 ± 223.85)		2 (15 ± 7.07)	
*Capillaria* spp.	1	40 (93.75 ± 181.76)	0.2	17 (35.29 ± 36.12)	0.3	14 (41.07 ± 39.96)	0.2
	2	25 (50 ± 53.52)	–	11 (25 ± 18.97)	–	4 (15 ± 7.07)	–
*D. caninum*	1	23	–	0	–	0	–
	2	0		0		0	
Taeniidae	1	5	–	0	–	0	–
	2	0		0		0	

^*^Statistically significant. *p* < 0.05

### Safety evaluation

No adverse events were observed throughout the course of the study.

## Discussion

Dogs can harbour multiple intestinal helminths infections [19], particularly hunting dogs, and are at higher risk of parasite infection in comparison to pet dogs. The rationale for this higher infection risk is owing to their outdoor access, roaming behaviour and active or accidental ingestion of eggs or larvae together with the paratenic host [10, 13]. Therefore, the administration of broad-spectrum anthelmintics or combinations of drugs is important to treat the polyparasitism in this specific canine population.

This study was conducted to re-evaluate the efficacy and safety of Drontal^®^ Tasty for treating intestinal helminths in naturally infected hunting dogs under field conditions in southern Italy. Since 1990, combinations of different anthelmintics, such as febantel, pyrantel pamoate and praziquantel have been used to target a broad spectrum of canine helminths, including nematodes and cestodes [36]. To provide veterinarians and owners with convenient means of treating and controlling helminth infections, a chewable tablet formulation was developed and approved in 1995 (Drontal^®^ Plus) after then in 2015 another commercial product (Drontal^®^ Tasty) was approved in Italy and then commercialized in 2016. The post-treatment anthelmintic efficacy of Drontal^®^ Tasty against *T. canis* was 99.7% and 100% at day 14 in the two study periods, the hunting- (P1) and the non-hunting-season (P2), respectively; the efficacy for *T. leonina* was 100% at day 14 (only in P1); the efficacy for Ancylostomatidae was 100% at day 14 in both P1 and P2, confirming the high efficacy of Drontal^®^ Tasty against these endoparasites.

The FECR against *T. vulpis* was variable with 88.6% and 99.8% at day 14 in P1 and P2, respectively. Similar results were obtained following anthelmintic treatment with chewable tablets containing afoxolaner + milbemycin oxime showing an anthelmintic efficacy ranging from 98.3% to 100% [14]. In P1D14 and in P2D14, 11 and 2 dogs were infected with *T. vulpis* eggs, respectively. Although the intensity of infection was quite similar (mean EPG count 14.5 at P1D14 versus 15.0 at P2D14), the higher number of infected dogs reported in P1 can be explained by several influencing factors, such as coprophagy, re-infection, the presence of immature stage, ingested and residual eggs. In fact, it should be also considered that failures in helminth control programs are often due to reinfection from contaminated environments and runs to which these type of dogs are exposed during the hunting season [27, 37]. Coprophagy, is a common behaviour in dogs, including hunting dogs, and can be attributed to various factors, including dietary deficiencies, behavioural issues and even potential inherited tendencies from wolf for maintaining den area free of faecal-borne intestinal parasites [38].

For these reasons husbandry practices and appropriate kennel management procedures may be required, particularly in kennel settings, to limit the re-infection. Factors such as kennel housing (e.g. individual versus group), the materials and structure of the kennels (e.g. concrete versus unpaved flooring) and effective faecal removal can significantly influence the persistence and transmission of parasitic stages in the environment [23]. In fact, owing to the natural habits and utilisation, re-infection with intestinal parasites is of high-risk among hunting dogs, wherefore it is important to implement regular internal parasite control measures. For example, it has been described that using anthelmintics that comprise of pyrantel pamoate have limited efficacy against adult whipworms. Further pyrantel as well as benzimidazoles have, due to their pharmacokinetic, poor efficacy against immature stages [39]. Consequently, following treatment, some larvae can develop into adult stage and excrete eggs [40]. According to the Companion Animal Parasite Control (CAPC), dogs infected with *T. vulpis* must be dewormed more than once with febantel–pyrantel–praziquantel at shorter intervals, with at least one treatment administered monthly for a period of three months [41, 42]. In fact, whipworms exhibit a reduced susceptibility to anthelmintics than other gastrointestinal parasites; often necessitating specific drug choices or multi-day/repeated treatment protocols to achieve high efficacy. While some newer anthelmintics or specific combinations can be effective in a single oral treatment against *T. vulpis*, the overall challenge of treatment is greater than for many other nematode infections [43]. However, in this study, the dogs only received a single treatment for study organizational reasons. Furthermore, the small sample size of *T. vulpis* positive tested dogs may have biased the overall efficacy results in P2 at D7 as all cleared on D14. Due to this, individual animal variations in FEC had a heavier impact on the overall efficacy results. Furthermore, in certain intestinal nematodes, such as *Ancylostoma*, FEC can transiently increase following the pharmacologically induced death of adult worms. This phenomenon is thought to be linked to a density-dependent fecundity mechanism, whereby the reduction in worm burden reduces intraspecific competition, allowing surviving females to temporarily increase their egg production [44]. This behaviour could also apply to *T. vulpis* and the higher EPG count in P2D7 could be explained by dying worms following treatment and the release of large amounts of eggs in the faeces. As the efficacy was 99.8% in P2D14, it is important to consider sufficient time between treatment and following faecal egg counts to evaluate treatment efficacies. In fact, a study showed that the anthelmintic efficacy of Drontal^®^ Plus Flavour increased from day 7 (95.6%) to day 21 post-treatment (100%) suggesting the use of a wider interval time for the evaluation of anthelmintic efficacy towards *T. vulpis* [22]. Nevertheless, the overall efficacy against *T. vulpis* was close to 90% (88.6%) in P1 (P1D14) and close to 100% (99.8%) in P2 (D14), confirming the overall high efficacy of Drontal^®^ Tasty in treating *T. vulpis*.

Drontal^®^ Tasty is not licensed (off label) for the control of *Capillaria* spp. In this study the percentage reduction against *Capillaria* spp. ranged from 42.1% to 84.5% during P1D14 and P2D14; according to another study showing that, although regular treatments with febantel, pyrantel and praziquantel were performed, *Capillaria* spp. eggs were reported in dogs suggesting their persistence in faeces [45]. Foxes are commonly infected by both *Capillaria aerophila* and *Capillaria boehmi* [46], as are hunting dogs that share environments with these wild animals, showing an increased risk of infection. This risk is further increased by coprophagy, a common habit among hunting dogs. The ingestion of infective eggs or larvae directly from the faeces of infected animals represents a direct and efficient pathway for acquiring these and other gastrointestinal or respiratory parasitic infections [8].

To the best of our knowledge there is no information regarding the impact of treatment intervals on helminth burden during hunting and non-hunting periods in dogs. In this regard, European Scientific Counsel Companion Animal Parasites (ESCCAP), and CAPC guidelines recommend that owners and veterinarians provide regular anthelmintic treatment to control all intestinal helminths in dogs on the basis of risk classification. The present study with its primary focus on hunting dogs to be followed during hunting and non-hunting seasons, demonstrated that hunting dogs should be treated with appropriate anthelmintics a minimum of two times per year. According to the ESCCAP guidelines, this frequency should be even more often for high-risk dogs, however, for many reasons, owners are not necessarily compliant, underscoring the importance of a minimum, appropriate anthelmintic treatment. This recommendation is crucial before hunting period, to improve the performance of dogs and reduce the contamination of parasite stages in hunting areas. Subsequent to the hunting period, the necessity for treatment is equally salient, to eliminate infection acquired during the hunt, as well as during outdoor activity. This is particularly crucial for dogs with extensive outdoor access, as their exposure mirrors that of hunting dogs in several critical ways. Such dogs frequently are in contact with wildlife or stray dogs, increasing the risk of infection through behaviours like coprophagy and ingestion of paratenic stage by predation [38, 47].

In Italy, the routine screening of hunting dogs for parasites remains notably infrequent, with only 4% of the dog owners that performed diagnosis [48]. Moreover, there is a lack of awareness among hunters regarding the necessity of regular parasitic surveillance and the targeted treatment, in particular against tapeworms. While data is missing, we do expect this ‘habit’ be no different in other European countries.

In this study, only owned dogs were included; thus, for ensuring their health and welfare status (including zoonotic risk for owners) all study dogs were treated, and no untreated control group was included [49] in contrast to the suggestion by Geurden et al. [50].

This study was not designed to assess the ease of administration; however, the easy administration and high palatability of the product, as suggested by veterinary practitioners, may be attributed to the addition of meat flavour to the formulation, thereby minimising stress to dogs during treatment.

The results of this study are consistent with those of other studies [51, 52], which demonstrated the high efficacy and safety of febantel–pyrantel embonate–praziquantel combination under field conditions, as no adverse events were observed during or after the study. For this reason, Drontal^®^ Tasty has proven to be easy to administer and highly manageable for the control of main intestinal helminths in hunting dogs.

## Conclusions

The aim of this study was to evaluate the efficacy of Drontal^®^ Tasty (febantel, pyrantel embonate, praziquantel) in Italy during the hunting and non-hunting season. The results of this study showed that this anthelmintic is safe and efficacious for the treatment of the major gastro-intestinal nematodes and cestodes in dogs. It significantly reduces parasite burdens, thereby enhancing overall dog health, and minimising risk of environmental contamination and the zoonotic potential. Preventive care for dogs should include regular deworming with licensed products, especially for hunting dogs with high-risk exposure to parasites.

## Supplementary Information

Supplementary Material1.

## Data Availability

Data supporting the main conclusions of this study are included in the manuscript.
