# Supplementary figures and images for: Field efficacy of Febantel, Pyrantel embonate and Praziquantel (Drontal® Tasty) against naturally acquired intestinal helminths of hunting dogs in southern Italy
Source: Parasit Vectors. 2025 Sep 24;18:377. doi: 10.1186/s13071-025-07027-z (PMC12462201; doi:10.1186/s13071-025-07027-z)

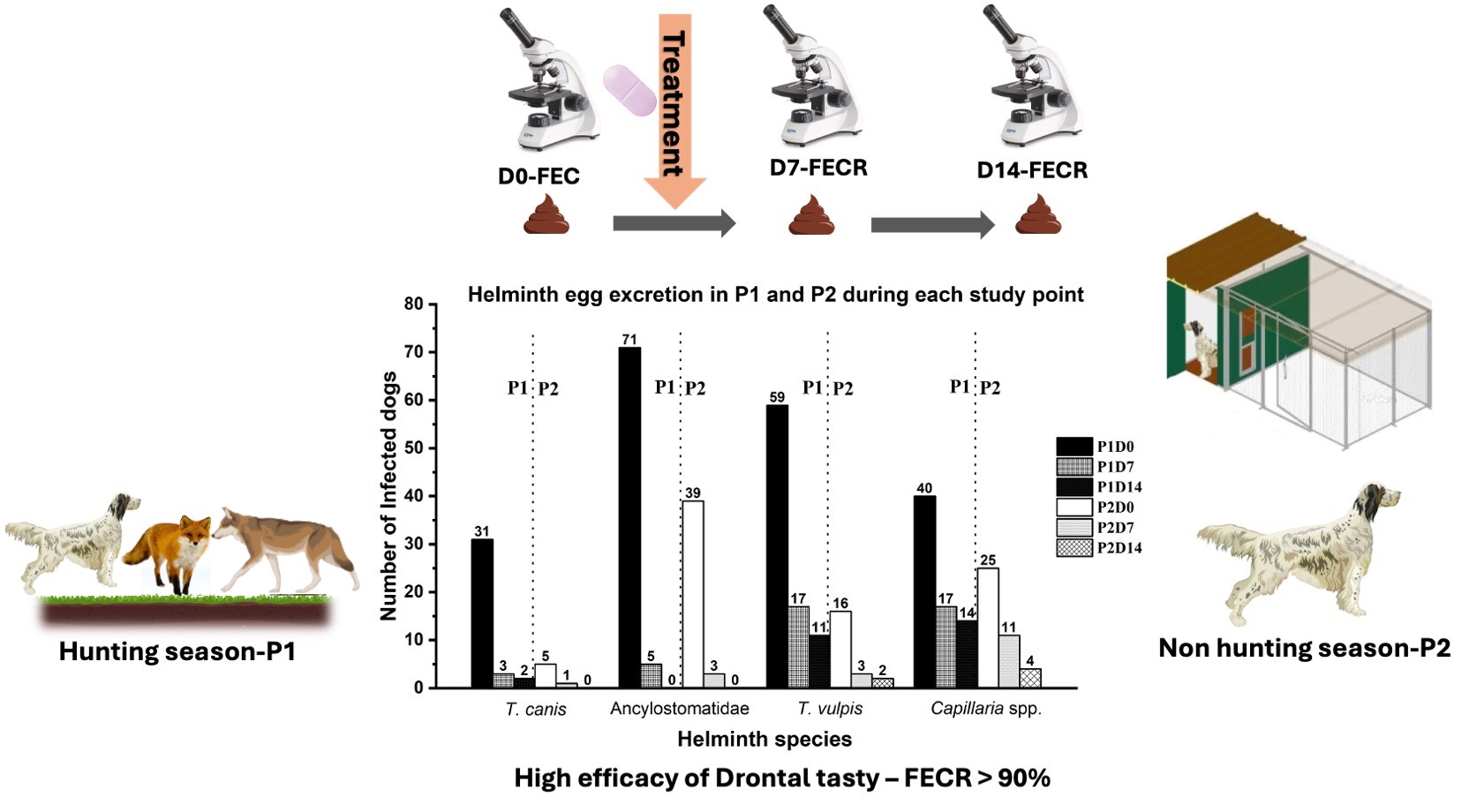

Supplement: Supplementary file 1 — Supplementary Material1. [file 13071_2025_7027_MOESM1_ESM.jpg]
